# Budget impact analysis of first-line treatment with pazopanib for advanced renal cell carcinoma in Spain

**DOI:** 10.1186/1471-2407-13-399

**Published:** 2013-09-02

**Authors:** Guillermo Villa, Luis-Javier Hernández-Pastor

**Affiliations:** 1 Departamento de Evaluación de Medicamentos y Gestión Sanitaria, GlaxoSmithKline España, Severo Ochoa, 2, 28760, Tres Cantos, Madrid, Spain

**Keywords:** Renal cell carcinoma, Kidney cancer, Pazopanib, Sunitinib, Markov models, Budget impact analysis, Cost analysis

## Abstract

**Background:**

Due to economic constraints, cancer therapies are under close scrutiny by clinicians, pharmacists and payers alike. There is no published pharmacoeconomic evidence guiding the choice of first-line therapy for advanced renal cell carcinoma (RCC) in the Spanish setting. We aimed to develop a model describing the natural history of RCC that can be used in healthcare decision-making. We particularly analyzed the budget impact associated with the introduction of pazopanib compared to sunitinib under the Spanish National Healthcare System (NHS) perspective.

**Methods:**

We developed a Markov model to estimate the future number of cases of advanced RCC (patients with favorable or intermediate risk) resulting either from initial diagnosis or disease progression after surgery. The model parameters were obtained from the literature. We assumed that patients would receive either pazopanib or sunitinib as first-line therapy until disease progression. Pharmacological costs and costs associated with the management of adverse events (AE) were considered. A univariate sensitivity analysis was undertaken in order to test the robustness of the results.

**Results:**

The model predicted an adult RCC prevalence of 7.5/100,000 (1-year), 20.7/100,000 (3-year) and 32.5/100,000 (5-year). These figures are very close to GLOBOCAN reported RCC prevalence estimates of 7.6/100,000, 20.2/100,000 and 31.1/100,000, respectively. The model predicts 1,591 advanced RCC patients with favorable or intermediate risk in Spain in 2013. Annual per patient pharmacological costs were €32,365 and €39,232 with pazopanib and sunitinib, respectively. Annual costs associated with the management of AE were €662 and €974, respectively. Overall annual per patient costs were €7,179 (18%) lower with pazopanib compared to sunitinib. For every point increase in the percentage of patients treated with pazopanib, the NHS would save €67,236. If all the 1,591 patients predicted were treated with pazopanib, the NHS would save €6,723,622 in 2013. Results were robust according to the sensitivity analysis.

**Conclusions:**

We developed a model that accurately reproduces the natural history of RCC and can be thus used in healthcare decision-making. When applied to the Spanish case, the introduction of pazopanib results in savings for the NHS, as a consequence of both reduced pharmacological costs and lower costs associated with the management of AE compared to sunitinib.

## Background

Renal cell carcinoma (RCC) is the most common kidney cancer [1] and accounts for approximately 3% of all cancers in males and 2% in females [2]. Advanced RCC has traditionally been a difficult to treat disease due to its inherent resistance to cytotoxic therapy, radiation or hormone therapy [3]. Prior to the advent of angiogenesis inhibitors, interferon alfa (INF-α) and interleukin-2 (IL-2) were the main therapies used for the treatment of advanced RCC, despite the significant toxicity and limited efficacy associated with their use [4,5].

Advances in the understanding of the molecular pathways of the tumor biology have enabled the identification of specific molecular targets for therapy, including the vascular endothelial growth factor (VEGF), platelet-derived growth factor (PDGF) and mammalian target of rapamycin (mTOR), what has led to the development of several drugs (sorafenib [6], sunitinib [7], bevacizumab (plus IFN-α) [8,9], temsirolimus [10] and everolimus [11]) that have substantially improved outcomes for RCC patients [12]. Pazopanib, a novel tirosinkinase inhibitor that targets VEGF, PDGF and stem cell factor receptor (c-Kit), is the latest drug approved for first line treatment of advanced RCC [13]. Pazopanib, sunitinib and bevacizumab (plus IFN-α) are recommended in the clinical guidelines for first-line treatment of advanced RCC in patients with favorable and intermediate risk [14-16].

COMPARZ (COMParing the efficacy, sAfety and toleRability of paZopanib vs. sunitinib) phase III clinical trial has evaluated the efficacy and safety of pazopanib compared to sunitinib in subjects with advanced RCC who had received no prior systemic therapy for advanced RCC. Pazopanib demonstrated non-inferiority to sunitinib in terms of median progression-free survival (PFS): 8.4 (95% CI: 8.3; 10.9) and 9.5 (95% CI: 8.3; 11.1) months, respectively (HR = 1.05 (95% CI: 0.90; 1.22 < 1.25)) [17].

Despite the current economic environment in which healthcare resources are scarce, to our knowledge, there is no published pharmacoeconomic evidence guiding the choice of one therapy over another as first-line therapy for advanced RCC in the Spanish setting. We aimed to develop a population-based model that describes the natural history of RCC and predicts the number of future cases of advanced RCC, so that it can be used in healthcare decision-making. We further aimed to use this model to analyze the budget impact (i.e. the financial consequence of adopting a new healthcare intervention [18]) associated with the introduction of pazopanib, compared to the current standard of care in Spain (i.e. sunitinib), in first-line treatment of advanced RCC under the Spanish National Healthcare System (NHS) perspective.

## Methods

### Epidemiology of advanced RCC in Spain

We modeled the annual number of patients diagnosed with or progressing to advanced RCC in Spain by means of a Markov model. Markov models are useful to represent random processes which evolve over time. With this methodology, a specific disease is described as a chain of different health states, and movements between those states over discrete time periods (cycles) occur with a given probability (transition probability). By running the model over a sufficient number of cycles, the long-term outcomes of the disease are obtained [19].

**Figure 1:**
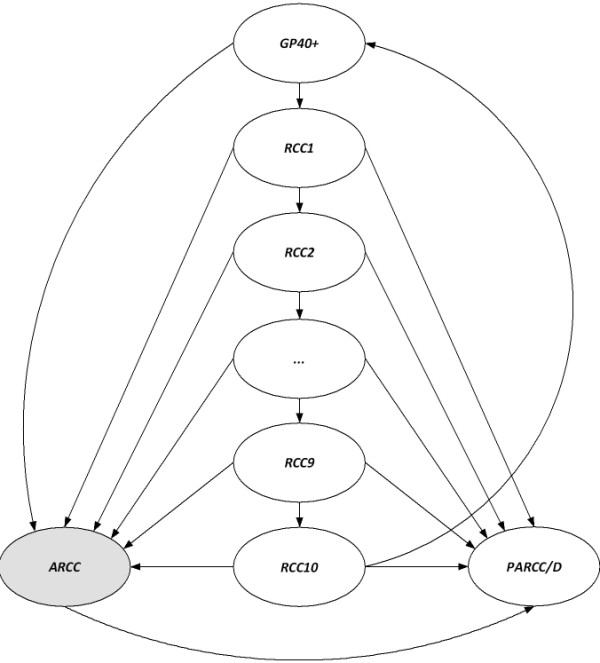
**Markov model diagram.** Detailed legend: *GP40+*: general population aged 40 and above, *RCC1* to *RCC10*: 10-year cohort of RCC prevalence, *ARCC*: advanced RCC patients, *PARCC/D*: post-advanced RCC patients or death

In this particular case, 13 health states were defined: *GP40+*: general population aged 40 and above; *RCC1 to RCC10*: 10-year cohort of RCC prevalence; *ARCC*: advanced RCC patients; and *PARCC/D*: post-advanced RCC patients or death (Figure 1). Since the probability of progression to advanced RCC after surgery for localized disease depends on time after the intervention [20], we used tunnel states (*RCC1* to *RCC10*) to incorporate this disease feature into the model. Tunnel states can be visited only in a fixed sequence. Their purpose is to apply to transition probabilities a temporary adjustment that lasts more than one cycle [21], thus overcoming the so-called “lack of memory” limitation of Markov chains. In order to allow patients with disease progression after surgery to be incorporated into the advanced RCC cohort, we carried out a simulation of the progression of RCC in the period 2003-2015, considering annual cycles. After 10 years, we assumed that patients treated for localized RCC were free of disease.

Model parameters were obtained from GLOBOCAN incidence figures, demographic data from the Spanish National Institute of Statistics and other evidence from the existing literature. Model parameters and their supporting references are presented in Table 1[7,13,20,22-26].

**Table 1:** Model parameters values and references: base case and sensitivity analysis

	**Base case**	**Sensitivity analysis**		**References**
**Parameter**		**Lower limit**	**Upper limit**	
Kidney cancer incidence 40+ (per 100,000)	19.8	17.82	21.78	[22]
RCC incidence (over kidney cancer incidence)	90%	85%	95%	[24,25]
RCC incidence at diagnosis (over RCC incidence)	20%	15%	25%	[24,25]
RCC mortality (localized disease)	1.66%	1.49%	1.83%	[23]
Transition to advanced RCC (year 1)	13.18%	11.86%	14.49%	[20]
Transition to advanced RCC (years 2 and 3)	4.57%	4.11%	5.02%	[20]
Transition to advanced RCC (years 4 and 5)	1.92%	1.73%	2.11%	[20]
Transition to advanced RCC (year 6 and 7)	1.64%	1.48%	1.81%	[20]
Transition to advanced RCC (year 8 to 10)	1.26%	1.13%	1.38%	[20]
Advanced RCC of favorable or intermediate risk	89%	80.1%	97.9%	[26]
Transition to post-advanced RCC	100%			[7,13]

### Cost analysis

We considered the annual pharmacological costs and also the costs associated with the management of adverse events (AE) for both pazopanib and sunitinib. Other costs, such as follow-up costs, were assumed to be equal for both treatments and thus were not taken into account. All costs were expressed in constant January 2013 Euro (€).

We considered 8 cycles of a 6-week treatment with either pazopanib (400 mg twice daily without interruption) or sunitinib (50 mg once daily for 4 weeks followed by a 2-week rest) per year. Ex-factory prices (VAT included) for pazopanib and sunitinib were obtained from the Spanish Council of Pharmacists database [27]. Patients who progressed on either pazopanib or sunitinib discontinued treatment. Based on progression-free survival Kaplan-Meier curves reported in COMPARZ [17], we assumed that, on average, patients would be on treatment with pazopanib or sunitinib 57% of the time within a year.

Incidence of AE for both pazopanib and sunitinib was obtained from COMPARZ [17]. In this analysis, we focused on AE with reported incidences (all grades) greater than or equal to 30% in either arm. Non-specific AE or those thought not to have contributed significantly to the overall costs (e.g. changes in hair color or taste alteration) were not taken into account. Laboratory abnormalities not associated with pharmacological treatment (e.g. creatinine increase or hypophosphatemia) were not considered. AE reported for pazopanib and sunitinib in COMPARZ are referred to median drug exposures of 8.4 months and 9.5 months, respectively. We assumed that reported rates of AE in clinical trials are equal to annual rates for the purposes of this analysis. Unit costs associated with AE management in the Spanish setting were taken from the literature [28,29] and expert judgment.

### Budget impact analysis

Budget impact analyses (BIA) are used to estimate the financial consequences of adoption of new healthcare interventions within a specific healthcare setting. A new healthcare intervention can either introduce savings into a healthcare system or put additional pressure on the healthcare budget due to modifications in the total population affected by a disease (e.g. better diagnostic tools), in the future population (e.g. preventive interventions that reduce disease incidence) or in the healthcare resources or drugs used to manage the disease [18].

We combined the estimated number of patients with advanced RCC provided by the Markov model and the cost analysis described above to simulate the budget impact resulting from the introduction of pazopanib, compared to sunitinib, under the Spanish NHS perspective. A temporal horizon of 3 years (2013–2015) was considered. Incremental annual costs were computed for any percentage of patients treated with pazopanib compared to sunitinib. Costs were discounted using a 3% annual rate.

### Sensitivity analysis

In order to test the robustness of the model, a univariate sensitivity analysis was undertaken. In this sensitivity analysis, one parameter is changed at a time and the new incremental cost is calculated. The lower and upper values of the model parameters used for this analysis are presented in Table 1.

## Results

**Figure 2:**
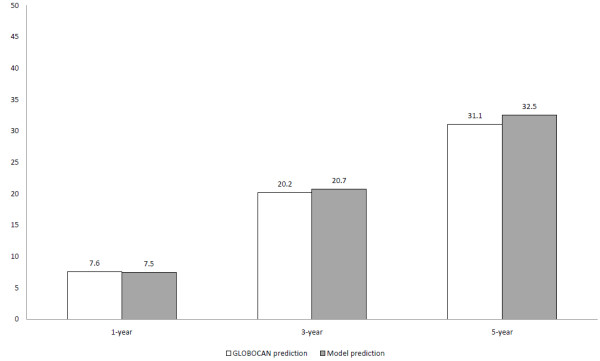
RCC adult prevalence (cases per 100,000), Spain 2013.

Adult RCC prevalence predicted by the model is as follows: 7.5/100,000 (1-year); 20.7/100,000 (3-year) and 32.5/100,000 (5-year). As can be seen in Figure 2, the model accurately matches GLOBOCAN reported prevalence figures for RCC (90% of kidney cancer prevalence [24]): 7.6/100,000, 20.2/100.000 and 31.1/100,000, respectively. These results validate the model externally in terms of its structure and the parameters chosen. The model predicts a total of 1,591 advanced RCC patients with favorable or intermediate risk in Spain in 2013. This figure is the result of the sum of the incident patients diagnosed with advanced disease within a year and those patients who relapse after surgery for the treatment of localized disease.

**Figure 3:**
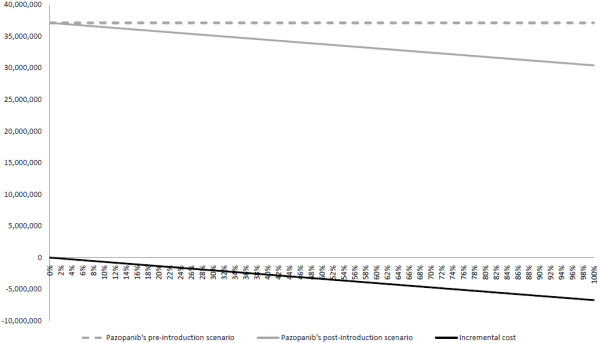
Overall annual costs, Spain 2013 (as a function of the % of patients treated with pazopanib).

Pharmacological costs per cycle for pazopanib and sunitinib were €4,046 and €4,904, respectively (Table 2). Annual (8 cycles) per patient pharmacological costs were 32,365€ and 39,232€, respectively. Costs associated with the management of AE were €662 and €974, respectively (Table 3). The overall annual per patient cost for pazopanib was €7,179 (18%) lower compared to sunitinib. The budget impact resulting from the introduction of pazopanib as a function of the percentage of patients treated is depicted in Figure 3. In 2013, a point increase in the percentage of patients treated with pazopanib compared to sunitinib would prevent the NHS from incurring an overall annual amount of €67,236. In the most efficient scenario, where all the 1,591 advanced RCC patients predicted by the model receive pazopanib, we estimate potential annual savings for the NHS of €6,723,622. Results for 2014 and 2015 are also presented in Table 2.

**Table 2:** Epidemiologic and economic results

	**Patients**	**Pazopanib**	**Sunitinib**	**Difference**	**Percent**
Per patient and cycle pharmacological costs		€4,046	€4,904	-€858	-17.50%
Per patient annual (8 cycles) pharmacological costs		€32,365	€39,232	-€6,867	-17.50%
Per patient annual costs associated with AE management		€662	€974	-€312	-32.03%
Per patient overall annual costs		€33,027	€40,206	-€7,179	-17.85%
**Year 2013**					
Advanced RCC at diagnosis	854				
Progressions to advanced RCC	934				
Advanced RCC (favorable or intermediate risk)	1,591				
Pharmacological costs		€29,350,968	€35,578,198	-€6,227,230	-17.50%
Overall costs		€30,404,210	€37,127,832	-€6,723,622	-18.11%
**Year 2014**					
Advanced RCC at diagnosis	866				
Progressions to advanced RCC	948				
Advanced RCC (favorable or intermediate risk)	1,615				
Pharmacological costs		€28,925,945	€35,063,000	-€6,137,056	-17.50%
Overall costs		€29,963,935	€36,590,195	-€6,626,260	-18.11%
**Year 2015**					
Advanced RCC at diagnosis	878				
Progressions to advanced RCC	962				
Advanced RCC (favorable or intermediate risk)	1,638				
Pharmacological costs		€28,483,391	€34,526,553	-€6,043,162	-17.50%
Overall costs		€29,505,501	€36,030,382	-€6,524,881	-18.11%

**Table 3:** Costs associated with the management of adverse events

	**Unit cost**		**Pazopanib**		**Sunitinib**		**Cost difference**
**Adverse event**	**Value**	**Reference**	**Incidence**	**Per patient cost**	**Incidence**	**Per patient cost**	
**Anorexia**		[28]		€4.97		€4.97	€0.00
Grades I and II	€13.43		36%		34%		
Grade III	€13.43		1%		3%		
**Diarrhea**		[28]		€127.12		€161.01	-€33.89
Grades I and II	€6.05		54%		49%		
Grade III	€1,376.13		9%		7%		
Grade IV	€6,171.42				1%		
**Fatigue**		[28]		€0.89		€1.02	-€0.13
Grades I and II	€1.62		44%		45%		
Grade III	€1.62		10%		17%		
Grade IV	€1.62		1%		1%		
**Hand-foot syndrome**		[29]		€23.30		€52.20	-€28.90
Grades I and II	€51.87		23%		38%		
Grade III	€189.41		6%		11%		
Grade IV	€1,165.50				1%		
**Hypertension**		Expert judgment		€33.72		€32.88	€0.84
Grades I and II	€16.88		30%		25%		
Grade III	€16.88		15%		15%		
Grade IV	€2,612.71		1%		1%		
**Nausea**		[28]		€52.28		€52.59	-€0.31
Grades I and II	€31.24		43%		44%		
Grade III	€1,942.30		2%		2%		
**Laboratory abnormality**							
**Anemia**		[28]		€65.77		€126.91	-€61.14
Grades I and II	€200.26		29%		53%		
Grade III	€261.70		1%		6%		
Grade IV	€507.46		1%		1%		
**ALT**		Expert judgment		€136.70		€79.77	€56.93
Grades I and II	€119.30		43%		38%		
Grade III	€236.16		15%		4%		
Grade IV	€2,498.64		2%		1%		
**Neutropenia**		[28]		€138.00		€248.60	-€110.60
Grades I and II	€356.78		32%		48%		
Grade III	€356.78		4%		19%		
Grade IV	€955.53		1%		1%		
**Trombocitopenia**		[28]		€78.83		€214.13	-€135.30
Grades I and II	€139.29		37%		56%		
Grade III	€449.54		3%		18%		
Grade IV	€1,380.29		1%		4%		
**Total**				**€661.58**		**€974.08**	**-€312.50**

**Figure 4:**
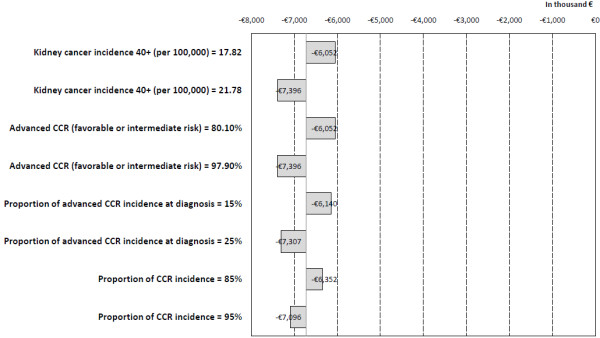
Sensitivity analysis: incremental cost resulting from univariate parameter changes (Tornado).

The univariate sensitivity analysis confirmed the robustness of the model. Among the model parameters, kidney cancer incidence, the proportion of advanced RCC patients with favorable or intermediate risk, the percentage of advanced RCC at diagnosis and RCC incidence were the most relevant. The incremental cost remained negative for any scenario considered, meaning that the introduction of pazopanib results in savings for the NHS (Figure 4).

## Discussion

Healthcare expenditure has drawn the attention of payers as well as of clinicians involved in oncologic care due to both the increased pressure on healthcare budgets as a consequence of the current economic environment and the relentless increase in healthcare spending as a portion of countries’ Gross Domestic Product over the past decades [30]. Anti-VEGF therapies for RCC are not an exemption and are subject to scrutiny from healthcare budget holders, pharmacists and oncologists alike. In this context, we sought to develop a model that describes the natural history of RCC, so that it can be applied to healthcare decision-making. To our knowledge, there are no published estimates of the future number of cases of advanced RCC in our country. We thus developed a time-dependent population-based Markov model to predict the future cases of advanced RCC and used this model to examine the budget impact associated with the introduction of pazopanib, compared to sunitinib, in the treatment of first-line advanced RCC patients with favorable or intermediate risk.

In order to effectively capture all the relevant costs and consequences, guidelines recommend BIA populations to be open [18,31], in the sense that individuals can enter or leave the population pool depending on whether they meet the criteria for inclusion (i.e. diagnosis of advanced RCC). This is in contrast with most Markov models in which populations are closed, with hypothetical patient cohorts being followed throughout a defined time horizon. Following a more realistic approach, we capture the changes in the advanced RCC population by means of a time-dependent population-based Markov model, based on the incidence of advanced RCC at diagnosis and on the likelihood of disease recurrence after surgery for localized disease. Patients leave the model when they experience progression during first-line therapy for advanced disease. Markov models have been used in other disease areas as well for this purpose [32].

The model accurately matches GLOBOCAN reported prevalence figures for RCC in Spain, providing evidence that it is able to reproduce the natural history of the disease and that it is therefore a reliable tool for estimating the future prevalence of advanced RCC based on RCC incidence. Moreover, the model results are robust as demonstrated by the sensitivity analysis performed. Even though this model includes Spanish-specific parameters (e.g. incidence rates and baseline populations), disease-specific parameters, such as the percentage of patients with advanced disease at diagnosis and the time-dependent probabilities of recurrence, have been obtained from the best available sources in the literature and are not country-specific. This model can be therefore easily transferred to other settings by simply replacing Spanish population estimates (publicly available from national statistics) and renal cancer incidence figures (publicly available from GLOBOCAN [22]) by country-specific data.

In our study, pazopanib results in considerable savings for the Spanish NHS, as a consequence of both reduced pharmacological costs and lower costs associated with the management of AE. Based on COMPARZ results, there are some AE that occur with a higher frequency with sunitinib (e.g. thrombocytopenia, anemia and neutropenia), while others seem to be more frequent with pazopanib (e.g. liver enzyme elevation) [17]. We thus included the costs associated with the management of AE for both drugs in order to account for such differences. Despite being very relevant for RCC patients [33], fatigue and hand-foot syndrome are not associated with a great increase in healthcare resource use or costly concomitant medications. They thus had a limited contribution to the difference in overall therapy costs in our analysis.

## Conclusions

We developed a time-dependent population-based Markov model that can be used to estimate the future number of cases of advanced RCC. We used it to undertake the BIA resulting from the introduction of pazopanib compared to sunitinib in the treatment of first-line advanced RCC under the Spanish NHS perspective. The introduction of pazopanib is cost-saving for the Spanish NHS, as a consequence of both reduced pharmacological costs and lower costs associated with the management of AE.

## Abbreviations

AE: Adverse events; BIA: Budget impact analysis; c-Kit: Stem cell factor receptor; €: Euro; IL-2: Interleukin-2; INF-α: Interferon alfa; mTOR: Mammalian target of rapamycin; NHS: National health system; PDGF: Platelet-derived growth factor; RCC: Renal cell carcinoma; VEGF: Vascular endothelial growth factor.

## Competing interests

GV and LJHP are employees of GlaxoSmithKline España.

## Authors’ contributions

GV and LJHP have equally contributed to the conception and design of the study, the analysis, acquisition and interpretation of data, and the drafting of the manuscript. Both authors have given approval of the final version of the manuscript.

## Pre-publication history

The pre-publication history for this paper can be accessed here:

http://www.biomedcentral.com/1471-2407/13/399/prepub
